# Assessment of child psychological attributes using strength and difficulties questionnaire for prediction of child behavior at first dental visit: a cross-sectional study

**DOI:** 10.1038/s41405-022-00103-x

**Published:** 2022-04-08

**Authors:** Sandra Moussa Anwar, Sara Ahmed Mahmoud, Mariam Mohsen Aly

**Affiliations:** Pediatric Dentistry and Dental Public Health, Faculty of Dentistry, Cairo University, Giza, Egypt

**Keywords:** Paediatric dentistry, Dental anxiety and phobia

## Abstract

**Introduction:**

Predicting child behavior before the first dental visit is considered an effective approach that enables the dentist to guide child behavior during the dental treatment.

**Aim:**

The present study aimed to detect psychological attributes of children seeking dental treatment for the first time using the strength and difficulties questionnaire (SDQ), evaluate actual child behavior during the dental treatment using Frankl Behavior Rating Scale, and test the association between these attributes and child behavior.

**Material and method:**

The study was conducted on 128 children aged between 4 and 7 years attending the outpatient Pediatric Dentistry clinic for the first time. Parents were asked to answer the SDQ, then a simple restorative treatment under local anesthesia was performed for children to evaluate their behavior using Frankl Behavior Rating Scale.

**Results:**

About 19.5% of the study sample were categorized as psychologically abnormal, where emotional and conduct problems were the most prevalent psychological attributes by a percentage of 22.7% and 20% respectively. A strong correlation was detected between the total difficulty score and child behavior.

**Conclusions:**

Psychological problems are common among children aged from 4 to 7 years and they also affect their dental behavior.

## Introduction

Child behavior is one of the fundamental challenges that face pediatric dentists owing to behavior management problems and their consequences. Therefore, prediction of child behavior before the first dental visit is considered an effective approach that enables the dentist to guide child behavior during the dental treatment [1].

Prediction of child behavior is based on the understanding of the factors that affect the child’s behavior in the dental setting, including age, maternal anxiety, and complexity of dental treatment. However, after adjustment of these factors, the association between the child’s psychological background and his behavior remains, which shows that psychological disorders have a significant influence on child behavior during dental treatment. Therefore, modern pediatric dentistry is paying huge attention to that psychological dimension [2, 3].

Psychological disorders are commonly classified into internalizing and externalizing problems, where internalizing problems are directed inward causing disorders within the individual including depression, anxiety, social isolation, or somatic complaints while externalizing problems are directed outward causing disorders with other people including aggressiveness, delinquency, or rule-breaking behavior [4, 5].

The strength and difficulties questionnaire (SDQ) is one of the most commonly used screening tools all over the world for the assessment of the child’s mental health status and is considered an effective tool in the prediction of child psychological disorders in psychiatry. It is used to measure five psychological attributes which include conduct, hyperactivity, emotional, peer problems, and pro-social behavior. Also, it classifies children according to their mental status into normal, borderline, and abnormal [6, 7].

Literature review reveals that limited studies investigated the relationship between child mental health and behavioral pattern of a child in the dental clinic. Therefore, this study aimed to detect psychological attributes of children seeking dental treatment for the first time using SDQ, evaluate actual child behavior during the dental treatment using Frankl Behavior Rating Scale, and test the association between these attributes and child behavior.

## Subjects and methods

### Sample size calculation

The sample size was calculated using Epi info for windows version 7.2 based on the results of Elhamid [8], who reported that the prevalence of emotional and behavioral symptoms among children was (20.6%). By adopting a confidence interval of (95%); the predicted sample size (n) was (128) cases.

### Eligibility criteria

The principal investigator screened Egyptian children with carious primary teeth attending the dental clinic for the first time with their parents to confirm adherence of the patients to the eligibility criteria.

The study included children aged 4–7 years old who visited the dental clinic with one of their parents with no previous dental experience and were diagnosed with pits and fissures caries at primary molars. Children who were medically compromised, whose parents refused to participate in the study, or who came to the diagnosis clinic with a dental emergency, were excluded from the study.

The current research was performed in compliance with the Helsinki Declaration. Ethics Committee of scientific research, Faculty of Dentistry, Cairo University approved the study with ID 19-6-8. This study was registered on clinicaltrail.gov under the title “Assessment of Child Psychological Attributes for Prediction of Child Behavior at First Dental Visit” with the identifier NCT03909243.

The purpose of the study, detailed description of the dental purpose, direct benefit to participants, and possible adverse events were explained to the participants and their parents in simple words. Signed informed consent was obtained from the parent and verbal assent was obtained from each child participating in the study.

This was a questionnaire-based cross-sectional study in which the principal investigator interviewed parents in the waiting area to answer the SDQ while all dental procedures were performed at the postgraduate clinics in Pediatric Dentistry and Dental Public Health Department, Faculty of Dentistry, Cairo University, Egypt by the principal investigator.

Using pre-prepared validated parent-version SDQ for children aged from 4 to 17 years old in Arabic, which was available online at www.sdqinfo.org. The principal investigator interviewed parents to answer a set of 25 questions with one of three answers (certainly true, somewhat true, not true).

Strength and Difficulties Questionnaire data were transformed to the SDQscore.org website for transcribing and scoring SDQ paper questionnaires. All psychological attribute scores, including emotional problems score, conduct problems score, hyperactivity score, peer problems score, prosocial score, total difficulties score, externalizing, and internalizing scores, were calculated. Inputting the results to this online system generated a set of final scores, along with categorization of these scores, as shown in Table 1. Although the term “ Abnormal” isn’t likely to be used today, it was utilized in the development of the original categorization and was followed for consistency.

**Table 1:** Categorization bands for SDQ scores for age 4–17 y.

SDQ scale	Normal	Borderline	Abnormal
Emotional problems score	0–3	4	5–10
Conduct problems score	0–2	3	4–10
Hyperactivity score	0–5	6	7–10
Peer problems score	0–2	3	4–10
Pro-social score	6–10	5	0–4
Externalizing score	0–7	8–10	11–20
Internalizing score	0–3	4–7	8–20
Total difficulties score	0–13	14–16	17–40

Dental treatment was scheduled to be performed in presence of the parents to make the child feel secure, but parents were instructed not to interfere by any means either verbally or by action. During the dental appointment, the child was asked to open his mouth wide and the buccal mucosa at the area of injection was dried and topical anesthesia was applied with a cotton bud for one minute, then the selected tooth was anesthetized using infiltration injection technique.

A simple class I restorative treatment was performed through complete caries removal using a round bur. Isolation of the tooth was managed by cotton rolls and saliva ejector followed by the application of etchant gel for 30 seconds then thoroughly rinsed in one step. A layer of the bonding agent was applied using a micro brush, then light-cured after 15 seconds and packable resin composite was used to restore the cavities. Child behavior was evaluated during the dental treatment by a trained investigator using the Frankl behavior rating scale (definitely negative, negative, positive, definitely positive) [9].

### Bias

All children fulfilling the inclusion criteria were included in the study until the completion of the study sample to eliminate any selection bias. To eliminate any performance bias, the questionnaire was completed by the parents outside the operating room, and the principal investigator who performed the dental treatment was blinded to the questionnaire answers.

### Statistical analysis

Statistical analysis was performed using the Statistical Package for Social Sciences (SPSS) version 18. Numerical data were summarized using mean and standard deviations. Categorical data were expressed as numbers and percentages and were analyzed using the chi-square test. Correlation between variables was performed using the Spearman test.

## Results

Children who participated in the present study aged between 4 to 7 years with a mean age of 5.34 ± 0.9 with equal distribution of male and female. Based on the total difficulty score collected, 19.5% (25 children) of the study population were lying in the abnormal category. Meanwhile, 21.9% (28 children) were categorized as borderline and 58.6% (75 children) were categorized as normal.

Emotional and conduct problems were found to have the highest prevalence among participants, with a percentage of 22.7% in the abnormal category for emotional problems and 20% in the abnormal category for conduct problems. Meanwhile, hyperactivity and peer problems came with a percentage of 9.5%, while pro-social problems were present only in 2.4% of the study population, as shown in Table 2.

**Table 2:** Distribution of different psychological attributes, internalizing, and externalizing problems among study population.

Psychological attributes	Categories	*N* (%)
Emotional	Normal	84 (65.6%)
	Borderline	15 (11.7%)
	Abnormal	29 (22.7%)
Conduct	Normal	74 (58%)
	Borderline	28 (22%)
	Abnormal	26 (20%)
Hyperactivity	Normal	107 (83.5%)
	Borderline	9 (7%)
	Abnormal	12 (9.5%)
Peer	Normal	104 (81%)
	Borderline	12 (9.5%)
	Abnormal	12 (9.5%)
Prosocial	Normal	114 (89%)
	Borderline	11 (8.6%)
	Abnormal	3 (2.4%)
Internalizing problems	Normal	35 (27.3%)
	Borderline	53 (41.4%)
	Abnormal	40 (31.3%)
Externalizing problems	Normal	82 (64.1%)
	Borderline	26 (20.3%)
	Abnormal	20 (15.6%)

A high prevalence of internalizing and externalizing problems was detected in this study sample, where 31.3% in the abnormal category of internalizing problems and 15.6% in the abnormal category of externalizing problems, as shown in Table 2.

According to the Frankl behavior rating scale, 8.6% (11 children) of the study population were extremely negative, 22.7% (29 children) were negative, 44.5% (57 children) were positive and 24.2% (31 children) were extremely positive.

A strong negative correlation was found between the emotional attribute score, the conduct attribute score, the hyperactivity attribute score, and child behavior. Also, a strong negative correlation was found between the internalizing score, the externalizing score, and child behavior. The total difficulty score showed a very strong negative correlation with child behavior, as shown in Table 3.

**Table 3:** Correlation between psychological attributes, internalizing, externalizing, total difficulty scores, and child behavior.

Attribute score	Spearman’s rho	Correlation with child behavior
Emotional	Correlation Coefficient	−0.422^**^
	*P* value	0.000
	Interpretation	Strong negative
Conduct	Correlation Coefficient	−0.566^**^
	*P* value	0.000
	Interpretation	Strong negative
Hyperactivity	Correlation Coefficient	−0.613^**^
	*P* value	0.000
	Interpretation	Strong negative
Peer	Correlation Coefficient	−0.353^**^
	*P* value	0.000
	Interpretation	Moderate negative
Prosocial	Correlation Coefficient	0.380^**^
	*P* value	0.000
	Interpretation	Moderate positive
Internalizing score	Correlation Coefficient	−0.509^**^
	*P* value	0.000
	Interpretation	Strong negative
Externalizing score	CorrelationCoefficient	−0.669^**^
	*P* value	0.000
	Interpretation	Strong negative
Total difficulty score	CorrelationCoefficient	−0.812^**^
	*P* value	0.000
	Interpretation	Very strong negative

** Correlation significant at *p* < 0.01.

Females showed more emotional problems than males by a percentage of 69% to 31%, respectively, with a *p* value = 0.029. Meanwhile, males showed more conduct problems compared to females by a percentage of 80.8% to 19.2% respectively with *p* value = 0.001, as shown in Table 4.

**Table 4:** Correlation between gender and psychological attributes, internalizing, externalizing, and total difficulty scores.

				Level		*P* value
			Abnormal	Borderline	Normal	
Emotional	Female	Count	20	9	35	0.029*
		%	69.0%	60.0%	41.7%	
	male	Count	9	6	49	
		%	31.0%	40.0%	58.3%	
Conduct	Female	Count	5	14	45	0.001*
		%	19.2%	50.0%	60.8%	
	male	Count	21	14	29	
		%	80.8%	50.0%	39.2%	
Hyperactivity	Female	Count	3	3	58	0.093 ns
		%	25.0%	33.3%	54.2%	
	male	Count	9	6	49	
		%	75.0%	66.7%	45.8%	
Peer	Female	Count	4	4	56	0.156 ns
		%	33.3%	33.3%	53.8%	
	male	Count	8	8	48	
		%	66.7%	66.7%	46.2%	
Prosocial	Female	Count	0	6	58	0.124 ns
		%	0.0%	54.5%	51.3%	
	male	Count	4	5	55	
		%	100%	45.5%	48.7%	
Externalization	Female	Count	8	6	50	0.002*
		%	12.5%	9.4%	78.1%	
	male	Count	12	20	32	
		%	18.8%	31.3%	50.0%	
Internalization	Female	Count	24	24	16	0.312 ns
		%	37.5%	37.5%	25.0%	
	male	Count	16	29	19	
		%	25.0%	45.3%	29.7%	
Total score	Female	Count	10	15	39	0.538 ns
		%	40%	53.6%	60.9%	
	male	Count	15	13	36	
		%	60%	46.4%	56.3%	

Significance level *p* ≤ 0.05, * significant, ns = non-significant.

Externalizing problems were found to be more evident in males than females by a percentage of 18.8% to 12.5% respectively for the abnormal category with a *p* value = 0.002. No statistically significant difference was found between both genders regarding the total difficulty score and the internalizing problems score with *p* value = 0.538 and 0.312, respectively, as shown in Table 4.

## Discussion

Assessing the child’s development, experiences, and current emotional state allows the dentist to develop a behavior guidance plan to accomplish the necessary oral health care. The importance of early detection of emotional and behavioral problems is being recognized worldwide. However, there has been little systematic research on childhood psychiatric disorders in developing countries [6, 10].

This study was held to assess psychological attributes among children aged between 4 and 7 years old as it was proved that at least 8–10% of children younger than five years experience clinically significant and impairing mental health problems, which include emotional, behavioral, and social problems. Children with these problems, as well as their families, experience distress, and can suffer substantially. They also demonstrate impairment across multiple domains, including dental treatment [11].

Based on the total difficulty score rating, 19.5% of all participating children were categorized as abnormal. This finding came in conformity with the one study that evaluated the prevalence of emotional and behavioral problems in Egyptian children using SDQ [8]. Also, this finding was in accordance with previous studies [12–14] that reported the prevalence of psychiatric problems among children from different community samples all over the world between 10% and 20%.

The high prevalence of psychological disorders, especially in developing countries, is strongly related to health and development concerns in young people such as low education levels, violence, malnutrition, and poor somatic health. It is also believed that the processes of urbanization and industrialization increase the risks for childhood emotional and behavioral problems [8].

In the Egyptian population, the higher prevalence of these psychological problems compared to other populations may be attributed to the public, professional, and political underestimation of child mental health problems in Egypt [8, 15].

About 22.7% of the study population were found to have emotional problems, 20% were found to have conduct problems, and 9.5% were found to have peer and hyperactivity problems, only 2.4% were having social problems which came in agreement with several studies [16, 17].

High rates of conduct problems in community samples can be justified by the poor quality education background, lack of discipline, and parental control. Meanwhile, the high prevalence of emotional problems is mainly because of parenting problems such as unstable home background, rejection by one or both parents, the intellectual expectation by the parents of a higher level than the child can achieve, or a physical defect which makes the child feels different from others [6, 18, 19].

About 31.3% of the study population were found to have internalizing problems, meanwhile, 15.6% were having externalizing problems. These results came in agreement with several studies [20, 21] that linked these findings to increased rates of single parenting, increased exposure to screen time, the internet, social media, and increased pressure within contemporary school settings.

Also, Samad et al. [22] reported a higher percentage of internalizing problems compared to externalizing problems who attributed that to the threat of parents achieving control over their children’s behavior.

According to the Frankl behavior rating scale, 8.6% of the children were extremely negative, 22.7% were negative, 44.5% were positive and 24.2% were extremely positive. Similar findings were found to be in line with previous studies, which can be explained because the roots of this behavior have a connection with the psychological state of participants toward dental treatment [2, 3, 23].

In consistence with previous studies [2, 3, 12], a strong negative correlation was detected between child behavior and each of emotional, conduct, and hyperactivity scores. This finding could be attributed to the fact that the child’s dental anxiety is linked to his psychological background. Children with high levels of dental anxiety were found to have aberrant personal characteristics, in addition to emotional and behavioral problems. This consequently affects their behavior during dental appointments, as children with these problems become more impulsive and show negative emotionality and general fearfulness [2, 3, 12].

Moreover, a strong negative correlation was found between child behavior and both internalizing and externalizing scores, which was confirmed by several studies [12, 24] that suggested using these two scores instead of the five scale scores may be more indicative for child behavior and more suitable in low-risk samples while retaining all five subscales when screening for a disorder

A very strong negative correlation was found between child behavior and total difficulty score. This strong correlation suggests that the total difficulty score is the best to predict the child’s behavior among other scores, and it was proved that it can be considered the most reliable score in screening psychological problems, especially in large samples. This can be attributed to the validity and internal consistency of this score, which is considered the most acceptable in comparison to the other two and five subscales scores [24, 25].

Girls were found to have more emotional problems by a percentage of 69% and boys had more conduct problems by a percentage of 80.8%. These results come in line with previous studies [26, 27] that reported females obtained higher scores in emotional and prosocial behavior; however, males tend to earn higher mean scores in conduct and peer problems.

These findings can be explained because certain behaviors are displayed more frequently or are more outspoken among males than females, and vice versa. Also, aggressive behaviors, including antisocial behavior and fighting in the school setting, were found to be more common in males, which were correlated with substance use, abuse, and inadequate parenting. Meanwhile, girls showed more early-onset childhood depression symptoms than boys, including shyness, withdrawal, hypersensitivity, and physical complaints [26, 28, 29].

Regarding internalizing and total scores, no statistically significant difference between both genders, but males seem to have more externalizing behavior problems than females where 18.8% of them were in the abnormal category and 31.3% were in the borderline category, which came in line with previous studies [4, 5]. These findings can be linked to escape from academic demands, access to attention, and difficulty in inhibiting emotional responses resulting from anger, frustration, and disappointment [30].

## Conclusions

From the results of this study, we can conclude that psychological problems are common among children aged between 4 and 7 years, specifically emotional and conduct problems, and the distribution of internalizing problems is twice as externalizing problems among those children.

A very strong negative correlation was found between total difficulty scores and child behavior. Therefore, the SDQ can predict the child’s behavior during the first dental visit enabling the dentist to identify psychological disorders and formulate a proper behavior guidance plan to accomplish child dental treatment.

Further studies with children of different age groups are needed to confirm the ability of this questionnaire to predict the child behavior for all children. Also. comparison between parent and teacher versions of the questionnaire is required to detect the more reliable model.
